# A Case of a Large Sub-retinal Abscess Secondary to Klebsiella pneumoniae Endophthalmitis in a Pyelonephritis Patient

**DOI:** 10.7759/cureus.4656

**Published:** 2019-05-14

**Authors:** Ismail Mohd-Ilham, Mustaqim Zulkifli, Maizan Yaakub, Rosiah Muda, Ismail Shatriah

**Affiliations:** 1 Department of Ophthalmology, Hospital Universiti Sains Malaysia, Kubang Kerian, MYS; 2 Department of Ophthalmology, Hospital Sultanah Nur Zahirah, Kuala Terengganu, MYS; 3 Department of Ophthalmology, School of Medical Sciences, Universiti Sains Malaysia, Kubang Kerian, MYS

**Keywords:** endophthalmitis, klebsiella pneumoniae, pyelonephritis, sub-retinal abscess

## Abstract

Endogenous endophthalmitis is an ocular emergency, with severe sight-threatening complications. We report a case of unilateral endogenous Klebsiella pneumonia endophthalmitis with a large sub-retinal abscess in a 39-year-old lady that developed four days after presentation with sepsis secondary to urinary tract infections and pyelonephritis. Despite immediate treatment with intravenous (IV) and intravitreal antibiotics, her eye condition deteriorated. A pars plana vitrectomy was performed, and the sub-retinal abscess was removed, followed by silicone oil tamponade. Subsequently, she regained her vision to 6/36 with complete regression of the intraocular inflammation and sub-retinal abscess.

## Introduction

Endogenous endophthalmitis is an ocular emergency in which pathogens reach the eye via the bloodstream, leading to possibly severe sight-threatening complications. It is relatively rare, accounting for 2% to 8% percent of all endophthalmitis cases in various studies [1]. In East Asian populations, Klebsiella was found to be responsible for approximately 80% to 90% percent of positive cultures and liver abscesses have been reported as the most common infectious origin [2]. In contrast, pyelonephritis, in association with endogenous Klebsiella pneumonia endophthalmitis, has rarely been described in the literature. This report aims to highlight a case of unilateral endogenous Klebsiella pneumonia endophthalmitis with a large sub-retinal abscess in a patient with pyelonephritis, with underlying uncontrolled diabetes.

## Case presentation

A 39-year-old lady with uncontrolled diabetes mellitus and a previous history of recurrent urinary tract infection secondary to right staghorn calculi was admitted to the medical ward due to sepsis with right pyelonephritis. Blood culture and sensitivity grew Klebsiella pneumonia. She was treated with intravenous cefepime. Unfortunately, on day four of admission, she developed sudden-onset reduced vision over the right eye associated with eye pain and redness.

**Figure 1:**
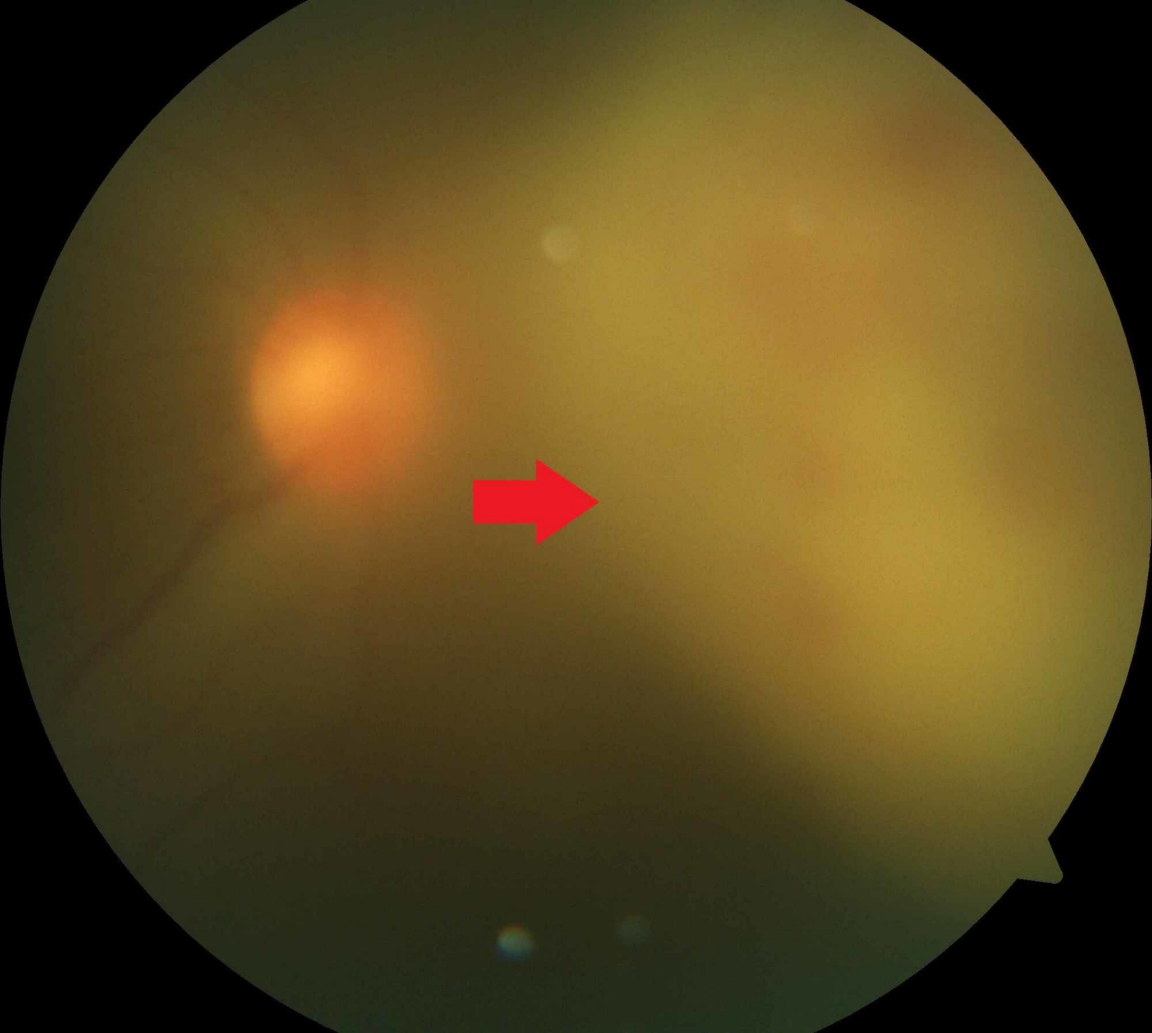
Large sub-retinal abscess on the nasal part of the retina, with severe vitritis

Her best-corrected vision was 6/60 in the right and 6/6 in the left eye. Right anterior segment examination showed injected conjunctiva with severe anterior chamber inflammation and hypopyon. Posterior segment examination revealed severe vitritis with a large sub-retinal abscess on the nasal part of the retina, about a four-disc diameter in size, extending from 1 o'clock to 5 o’clock with inferior exudative retinal detachment (Figure 1).

The left eye examination was unremarkable, with no apparent diabetic retinopathy. B-scan ultrasonography demonstrated a sub-retinal hyperechoic lesion on the nasal side of the globe, consistent with a sub-retinal abscess.

The diagnosis of right endogenous endophthalmitis was made. An intravitreal tap and injection of antibiotics (vancomycin 2 mg/0.1 ml and ceftazidime 2 mg/0.1 ml) was done immediately and intravenous ciprofloxacin, along with eyedrops (cefuroxime, gentamicin, and dexamethasone), were given. Liver ultrasound was performed and showed no evidence of liver abscess.

**Figure 2:**
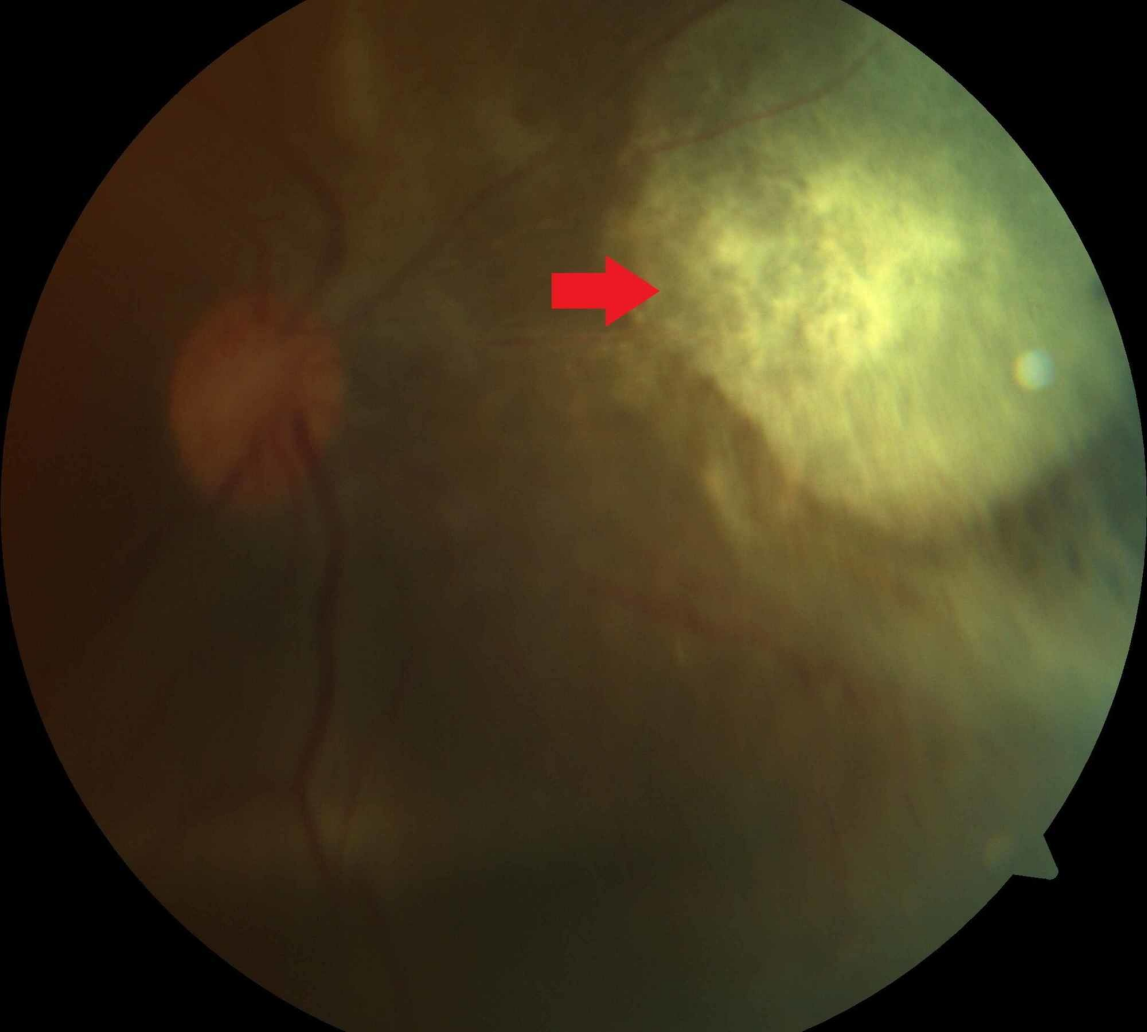
Resolved sub-retinal abscess and intraocular inflammation

Despite intensive systemic and topical antibiotics, her right eye condition worsened and her vision dropped to counting fingers. She finally underwent vitrectomy with silicone oil tamponade two days later. Postoperatively, the patient responded well to treatment, and her vision was gradually improved. At the three-month follow-up, her right best-corrected visual acuity was 6/36, and there was complete resolution of intraocular inflammation and the sub-retinal abscess (Figure 2).

## Discussion

Endogenous endophthalmitis (EE) is an ongoing diagnostic and therapeutic dilemma for ophthalmologists, as it is relatively rare, often presents like uveitis, and requires a high index of suspicion for prompt diagnosis and treatment [1,3-5].

The major risk factor that contributes to endogenous endophthalmitis was immunocompromised state, including prolonged corticosteroid use, malignancy, end-stage renal or liver disease, post-organ transplant, and diabetes mellitus. Illicit intravenous drug use, indwelling catheter, or dental procedures were also reported as significant risk factors [4-8]. EE has been reported in healthy individuals in an extraordinary case [3]. Lee et al. (2012), in their case series covering over 15 years of clinical experience in Korea, demonstrated that 52 patients (65%) had one or more sources of infection, with liver abscesses being the most common (20 cases, 25%). Urinary tract infection contributed only 5% of cases. Thirty out of 62 cases (48.4%) had Klebsiella pneumonia [4]. However, Muda et al. (2018) and Michael et al. (2018) reported a higher percentage of urinary tract infections as the source of bacteremia in endogenous endophthalmitis, 17.5% and 29.4%, respectively [5-6].

The predisposing factors identified in this patient were uncontrolled diabetes mellitus and recurrent urinary tract infection secondary to staghorn calculi for eight years. Moreover, she refused further surgical intervention and defaulted subsequent follow-up at the surgical clinic for her renal problem. Klebsiella pneumonia was isolated from the blood but vitreous culture turned out to be negative. This could be due to the partial treatment since she was started with a systemic antibiotic before she developed endophthalmitis.

Generally, the visual outcome of endogenous endophthalmitis is usually poor due to early and extensive retinal involvement. Virulent causative organisms, poor host defense, misdiagnosis leading to delayed treatment, inadequate treatment, inappropriate therapy, and the occurrence of complications such as panophthalmitis, are associated with poor prognosis [7-8]. Surprisingly, the visual outcome of this patient was relatively good because she was referred on the same day to an ophthalmologist when having visual symptoms, early diagnosis, immediate intravitreal antibiotics, and early vitrectomy. Apart from that, the location of the sub-retinal abscess also played a major contributing factor for better visual outcome. The sub-retinal abscess was located on the nasal side, which is away from the posterior pole and optic disc.

## Conclusions

Pyelonephritis should be considered one of the sources of infection in Klebsiella pneumoniae endophthalmitis in a diabetic patient. The visual outcome has been extremely poor in most cases. However, treatment could be successful with early diagnosis, intravitreal systemic antibiotics, and early vitrectomy.
